# Impact of respect, equity, and leadership in brain health

**DOI:** 10.3389/fneur.2023.1198882

**Published:** 2023-08-08

**Authors:** Faheem Arshad, Jonathan Adrian Zegarra-Valdivia, Caroline Prioleau, Victor Valcour, Bruce L. Miller

**Affiliations:** ^1^Global Brain Health Institute, University of California, San Francisco, San Francisco, CA, United States; ^2^National Institute of Mental Health and Neurosciences, Bengaluru, India; ^3^Department of Human Medicine, Universidad Señor de Sipán, Chiclayo, Peru; ^4^Department of Neurology, Memory and Aging Center, Weill Institute for Neurosciences, University of California, San Francisco, San Francisco, CA, United States

**Keywords:** equity, brain health, leadership, respect, values

## Abstract

Respect is a feeling of admiration for someone. It forms one of the core values of the Global Brain Health Institute (GBHI), which strives to protect the world’s aging populations from threats to brain health. These values guide us as we advocate for reducing the global impact of dementia. By taking a values-based approach to brain health, we can drive global changes for millions of people. Respect fortifies gratitude and embraces diversity. Philosophical discussions of the ideas support the assertion that respect is crucial in everyday conversations and actions as well as in personal, social, political, and moral spheres. No one can become a leader unless they genuinely respect and care about the success of each team member. Diversity, equity, and inclusivity form the fundamental cornerstones of respect. Understanding this core value of respect will ensure altruistic behavior among the leaders that may help mitigate racism, cultural insults, gender discrimination, stigmatization, religious hatred, and, worst of all, poor leadership abilities that have been the disconcerting examples of disrespect in recent years. We present the underlying neurobiology of respect and its impact on equity and leadership.

## 1. Introduction

Respect, also called esteem, is a feeling of admiration towards someone. Philosophers define the central concept of respect in various ways as a mode of behavior, a form of treatment, a valuing, a type of attention, a motive, an attitude, a tribute, a principle, a moral virtue, or an epistemic virtue (1). It is one of the core values of the Global Brain Health Institute (GBHI) that strives to protect the world’s aging populations from threats to brain health. These values are known by the acronym “A FORCE” and stand for authenticity, fairness, openness, respect, courage, and empathy. These values are connected.

Authenticity assumes a genuine foundation for the initiation of any action. This action may demand fairness in the process, highlighting the need for transparency, equity, and openness. This underlies the curiosity required for evidenced-based change, and this cannot be achieved without respect which fortifies gratitude and embraces diversity. But, to accomplish this, one must be courageous to take risks. And finally, at the heart of community-informed change is the core value of empathy, the ability to feel what others are feeling as if you are feeling it yourself. These values can guide in advocating for reducing the impact of dementia. A transformation may be kindled for millions of people globally by using this values-based approach to brain health.

As we navigate through unprecedented times, human behaviors, like COVID-19 are propagated unpredictably. People’s respect can be fickle. “A reputed selfless King was worshipped and respected in a village, and songs were sung in his honor. However, one day everything changed after a young girl in the village became pregnant and gave birth to a child. When asked about the father, she said he was the King’s child. How long does it take for respect to become disrespect and admirers to become enemies? The mind waits for a chance; the day admiration ends, condemnation begins. The people of the village attacked the King.^1^”

## 2. Neurobiology of respect

Respect is central in many disciplines, including biomedicine, ethology, philosophy, and ethics. It has the distinctive property of acting as a “verb and noun,” simultaneously activating “action and feeling” in human behavior (2). Respect is a positive social emotion associated with a deep sense of admiration. When recognizing laudatory behavior in another person, Kant suggests (3), “Act in such a way that you treat humanity, whether in your own person or the person of any other, never simply as a means but always at the same time as an end,” referring to the inherent worth that shapes our identity and interactions with others.

The complexity of this value can be traced back evolutionarily through dominance hierarchies seen in animal societies, including our closest ancestors, the great apes (4). Related concepts, such as respect for ownership, are lost in young children and only found in children with object-subject conceptualization and theory of mind (ToM) (5, 6). In an experimental study, respect for ownership was found in 4 years-old children in more than 85% of the trials (6), and it is similar across societies in children from 4 to 7 years old (7, 8). Younger children with non-development in ToM and object-subject conceptualization may fall in recognition of ownership and respect. Respect may be supported by the theory of mind, empathy process development, and social hierarchy (9, 10), which may refer to the social brain and prefrontal cortex development (11). Besides, different studies have consistently identified several brain regions as involved in social dominance, including the amygdala, the hippocampus, the striatum, the intraparietal sulcus, and the prefrontal cortex (10) as neuronal networks involved in the social brain, emotional and reward pathways.

Positive feedback activates the ventral tegmental area (VTA) and its projections to the striatum, causing dopamine to be released, which is responsible for positive feelings, optimism, and sociability (12). We are more likely to feel valued and respected with its release. In turn, we become motivated to continue performing/behaving well to gain recognition and to pursue connections with others to achieve common goals (13). Respect and disrespect have distinct components that are cognitive and emotional. At the cognitive level, we construct values we admire in ourselves and others. Simultaneously there are other features that we shun in our semantic appraisal network. The feelings have less to do with rational, cognitive, and higher-level thinking in the neocortex. Instead, these emotions are linked to the limbic system and its connections, including the amygdala, orbitofrontal cortex, hippocampus, striatum, and cingulate—areas involved in emotional, motivational, and reward processes.

Feeling disrespected activates the amygdala, a part of the brain’s system that processes strong emotions (14). Even if the person performing the behavior believes they are acting perfectly respectfully, it is perceived as a threat. In contrast to the support received in a social context, the amygdala also mediates the relevance of emotion preceding the significance of the action (15). Aside from this structure, the hypothalamus mediates the self-preservation instincts and the fight-flight-freeze response, which is triggered in the peri-aqueduct grey area and transmitted to the hypothalamus in less than a second. It regulates autonomic function, stress response, temperature, and other essential body processes. When one is respected, hormones such as oxytocin (16, 17) and serotonin are released in the creation of bounding; in contrast, when one is disrespected, hormones such as adrenaline, cortisol, and norepinephrine are released (18). Nonetheless, further research is need to elucidate completely the neurobiology behind values and respect.

### 2.1. Neuroanatomical localization of respect

So, where in the brain does respect reside? When appreciating exceptional behavior, for example, positive social emotions are typically felt as a way of showing respect. Semantic memory is required for this process. Social semantic information is stored in the anterior temporal lobe (ATL) (19). ATL activity is modulated by semantic knowledge’s conceptual features because it necessitates the appraisal of exceptional behavior, as well as the person as a whole; respect activates the ATL. On the other hand, admiration is associated with a person’s ideal behavior. In their study, Nakatani et al. (20), observed that appraisal ratings for a person’s behavior were higher in admiration-related vignettes. In respect-related vignettes, however, those for the person were higher. The intensities of admiration and respect differentially modulated brain activity in a part of the left ATL. Other significant areas include the medial, orbitofrontal, temporal, and cingulate cortex, which are related to understanding and predicting other people’s feelings, ideas, and behaviors, representing the theory of mind (5, 21, 22). This process can potentially alter social interactions by mediating respect behaviors, social needs, emotional reactions, and normative expectations. In another study, Nakatani et al. (20) observed an association of respect and empathic concern with reduced gray matter in the left ATL. Mediation analysis revealed that respect directly affected the gray matter volume when empathic concern was a mediation variable.

On the other hand, is not surprising that prefrontal areas might be involved in respect processing, for example, due to balance decision-making processes through “good or bad” options, representation of reward values, non-rewards and punishment, as well as social relevant information (23, 24). Nonetheless, more research data and new experimental paradigms are needed to completely elucidate brain areas and networks involve in highly hierarchical brain function related to moral values.

## 3. Diversity in respect

Racism, cultural insults, gender discrimination, stigmatization, religious hatred, and poor leadership abilities have been disconcerting examples of disrespect in recent years. All of these have contributed to inequity in the world’s different global communities, integrating disrespect, prejudice, and power, whether expressed quietly or actively, knowingly or unconsciously. Respect is vital in terms of equity and leadership. Unlike egoism, “altruism” is a powerful construct based on a blurred line between the self and the environment, manifesting as a sense of connectedness (25). Altruistic behaviors studied in infants have been shown to maintain and foster future altruistic behaviors throughout development and into adulthood, inadvertently imprinting the moral value of respect globally (26). Altruism appears to be demonstrated through prosocial behaviors, measured using self-reported scales that precisely measure altruistic behavior. The neural correlates of altruistic behavior in the brain include the regions within the mentalizing network, such as the medial prefrontal cortex and temporoparietal junction; reward regions, including the ventral tegmental area, striatum, specifically the nucleus accumbens, and anterior cingulate cortex, and areas of emotional salience network including the dorsolateral prefrontal cortex, insula, and amygdala (27, 28).

When confronted with a predicament, the emotional brain reacts more quickly than the rational brain. This is how humans have evolved and survived, which is critical for leaders to understand. Leaders must proactively aim to rewire their brains to appreciate “others,” or those who are not like them, in order to ensure equity among those they lead. Inextricably related are respect and leadership. Altruistic acts foster a sense of respect and equity, both of which are critical elements of good leadership. Outstanding leadership, in turn, generates a climate of respect and high expectations, encouraging everyone to accomplish their best. Leaders must be humble enough to acknowledge that no one has solved the numerous complex problems and that solving them will always be difficult owing to intractable ambiguity. On the other hand, being receptive to novel and unconventional ideas may aid in navigating such problems and identifying the best possible solutions. Recognizing differences, demonstrating cultural humility, and treating others with respect are all essential to achieving objectives.

## 4. The final verdict

“The girl expressed regret for making false accusations. The villagers questioned the King, ‘Why did not he refute this at the outset?’ The King said, ‘What difference would it have made? The child must belong to someone. People would have relished defaming one more individual if they had the opportunity.’ If the King had been concerned about their condemnation, he would also have been concerned about their respect. He had become indifferent to people’s behaviors by dropping the idea of good and bad because the thinking makes it so (see footnote 1).”

Indifference to good and bad becomes evident in the activities of real-life practitioners due to the dehumanization in medicine (29). Nonetheless, integrating “A FORCE” concepts in general practice may help “re-humanize” patients (30). Positive psychology and positive psychiatry, as a humanistic movement, could support this process through positive interventions based on the total respect of patient’s optimism, wisdom (31), and kindness (32). From our point of view, positive psychology and psychiatry’s principles are needed to extend implemented in future practitioners’ curricula through promote soft skills sometimes lost in clinic practice. For instance, a humanistic perspective base on values might improve brain health outcomes, even though more research data are needed to validate and extend these practices worldwide to impact overall health care (33). Moreover, these approaches need to be extended even more in society, for example, through regional and global initiatives that impulse multi-level brain health-directed policy agendas, focus to augment sustainability of democratic societies through brain capital, equity, mental health and resilience (34), which not only goes in line with “A FORCE” concepts, but also promotes brain health, altruistic behaviors and democracy (35).

Respect and altruistic behaviors are among the ultimate solutions to global inequity, mediated by selfless leadership skills, one of the core values at the Global Brain Health Institute (GBHI). The acronym for “RESPECT” highlights the construct of altruism: remain calm, encourage others, stay positive, politeness, embrace differences, consider consequences and, more importantly, think before you speak.

## 5. Limitations and future directions

The study of the neurobiology of values, including respect, faces limitations that must be considered. Firstly, the definition and nature of the respective value can pose challenges when operationalizing the concept and conducting subsequent research. Additionally, incorporating novel methodologies such as neuroimaging (e.g., fMRI, EEG, MEG) holds promise for a deeper understanding of the neurobiological underpinnings of values and respect.

Exploring respect as a value raises intriguing questions from neurobiological and philosophical perspectives that remain open for further investigation. We are still far from obtaining a definitive answer regarding the specific neuronal circuits involved. However, progress can be made by studying human psychology and cognitive neuroscience through experimental procedures and neuroimaging techniques. This interdisciplinary approach will contribute to advancing our knowledge of how different brain areas and neural networks are implicated in the concept of values.

Overall, respect is a complex emotion that allows us to interact in human social situations successfully. But how do we regulate respect in the brain? Is respect influenced subconsciously or consciously by the behaviors of those around us? If so, how much influence does it wield? And how does neural connectivity make this possible? These questions are difficult to fully answer, despite our current understanding of the human brain. The main aim of this manuscript was to generate hypothesis in this field. However, this area is raw, and the summary of the progress needs to be interpreted with caution. Thus, more studies are required for generating evidence with regard to understanding the neurobiology of respect and whether public policy interventions could help boost population respect.

## 6. Conclusion

In conclusion, integrating cognitive and social neuroscience with new methodologies represents a compelling research area within social cognitive neuroscience. Numerous studies have identified neurobiological markers corresponding to values, but it is not enough. Future research should aim to explore the neurobiological mechanisms underlying values in general, including respect, and investigate specific neural networks associated with these values. Furthermore, understanding the significance of these networks in clinical practice will enhance our comprehension of how social factors impact mental health.

## Data availability statement

The original contributions presented in the study are included in the article/supplementary material, further inquiries can be directed to the corresponding author.

## Author contributions

FA and J-ZV: conceptualization, writing the original draft, reviewing, and edition of final draft. BM, VV, and CP: writing, reviewing, and editing the revised draft. All authors contributed to the article and approved the submitted version.

## Conflict of interest

The authors declare that the research was conducted in the absence of any commercial or financial relationships that could be construed as a potential conflict of interest.

## Publisher’s note

All claims expressed in this article are solely those of the authors and do not necessarily represent those of their affiliated organizations, or those of the publisher, the editors and the reviewers. Any product that may be evaluated in this article, or claim that may be made by its manufacturer, is not guaranteed or endorsed by the publisher.

## Footnotes

^1^Regional history.
